# Exploration of proper heating protocol for injectable horizontal platelet-rich fibrin gel

**DOI:** 10.1186/s40729-022-00436-0

**Published:** 2022-09-13

**Authors:** Xijiao Zheng, Xiang Yan, Kai Cheng, Mengge Feng, Yulan Wang, Bing Xiao

**Affiliations:** 1 Xiantao First People’s Hospital Affiliated to Yangtze University, Xiantao, China; 2 State Key Laboratory Breeding Base of Basic Science of Stomatology (Hubei-MOST) and Key Laboratory of Oral Biomedicine, Ministry of Education, School and Hospital of Stomatology, Wuhan University, Wuhan, 430079 China

**Keywords:** Blood, Platelet-rich fibrin, Gel, Horizontal centrifugation, Tissue regeneration

## Abstract

**Purpose:**

Platelet-rich fibrin (PRF) has been proposed as promising biomaterials with the advantages of host accumulation of platelets and leukocytes with entrapment of growth factors and fibrin scaffold. However, limitations including fast resorption rate (~ 2 weeks) restricts its clinical application. Recent studies have demonstrated heating treatment can prolong PRF degradation. Current published articles used the method of 75 °C for 10 min to obtain longer degradation, while few studies investigated the most suitable temperature for heating horizontal PRF. Our present study was to discover and confirm the optimum temperature for heat treatment before obtaining H-PRF gels by investigating their structure, mechanical properties, and bioactivity of the H-PRF gels after heating treatment.

**Methods:**

In the present study, 2-mL upper layer of horizontal PRF was collected and heated at 45 °C, 60 °C, 75 °C, and 90 °C to heat 2-mL upper layer of horizontal PRF for 10 min before mixing with the 2-mL lower layer horizontal PRF. The weight, solidification time and the degradation properties were subsequently recorded. Scanning electron microscopy (SEM) and rheologic tests were carried out to investigate the microstructure and rheologic properties of each H-PRF gel. The biological activity of each H-PRF gel was also evaluated using live/dead staining.

**Results:**

H-PRF gel prepared at 75 °C for 10 min had the fast solidification period (over a tenfold increase than control) as well as the best resistance to degradation. The number of living cells in H-PRF gel is greater than 90%. SEM showed that H-PRF gel becomes denser as the heating temperature increases, and rheologic tests also revealed that the heat treatment improved the mechanical properties of H-PRF gels when compared to non-heated control group. Future clinical studies are needed to further support the clinical application of H-PRF gels in tissue regeneration procedures.

**Conclusions:**

Our results demonstrated that the H-PRF gel obtained at 75 °C for 10 min could produce a uniform, moldable gel with a short time for solidification time, great rheologic behavior and, high percent of live cells in PRF gel. A promising use of the commonly utilized PRF gel was achieved facilitating tissue regeneration and preventing degradation.

## Background

Over the past few decades, platelet concentrates have been developed and been widely used in dental practice, facial esthetics, sport medicine, osteoarthritis, et al. Platelet concentrates became popular biomaterial as they can be prepared easily by collecting patient’s own peripheral blood and centrifugation, which is a simple and low-cost protocol by single centrifugation without the addition of anticoagulants. PRF contains live cells, abundant growth factors (GFs) [1] and three dimensional fibrin network, showing promising effects in tissue regeneration [2–4]. Among different platelet concentrates, Horizontal platelet-rich fibrin (H-PRF) is proposed and shown to contain more growth factors by a novel horizontal centrifugation after blood collection which is verified to contain a greater number of live cells distributed more evenly in the final products. H-PRF is found to show better performance in tissue regeneration. Centrifugating PRF in plastic centrifuge tubes allowed PRF to be prepared as a liquid form and used in injectable way [5]. Liquid PRF can be used in various tissue regeneration scenes, such as facial esthetics, joint injection or combination use with bone substitutes. Especially in facial regeneration, liquid PRF can act as a natural filler and release much growth factors to induce tissue regeneration compared with other artificial biomaterials. What’s more, the fibrin, fibronectin, and other extracellular matrix proteins contained in PRF are beneficial for inducing cell proliferation and differentiation in dermis.

One of the most important limitations of PRF is the short local persistence as well as low mechanical strength when used for tissue regeneration. Several attempts have been made to improve the stability of PRF at the surgical site or as facial fillers by changing the centrifugation protocols and by combining PRF with other biomaterials to improve bone and soft tissue healing by changing the biodegradability of the membrane [6–8]. However, adding exogenous biomaterials in PRF might hinder their biosafety. Recently, several studies proposed a new protocol to extend the degradation time of PRF in vivo which utilizes heat treatment of the plasma layer after centrifugation [9–12]. Although the growth factor levels found in PRF would normally be lost in standard heating protocols, methods have recently been proposed to reincorporate the heated plasma layer with Liquid PRF containing cells extracted from buffy-coat layers, to increase the bioactivity of the final PRF mixture. This novel method has been shown to be solid, stable and opaque, releasing high levels of growth factors for up to 10 days or more [9, 11].

Thus, the hypothesis of the present study is that there is a most proper temperature for heating the upper layer of H-PRF before mixing with buffy coat, which can best improve the degradation time and mechanical properties of H-PRF gel without losing much bioactivity for utilizing as injectable fillers. To explore the proper temperature for heating H-PRF, a series in vitro experiments were designed by reporting the changes of degradation time, rheological properties and cell viability of H-PRF gels after heat treatment. First, 2-mL upper layer of horizontal PRF was collected and heated at 45 °C, 60 °C, 75 °C, and 90 °C for 10 min before mixing with the 2-mL lower layer horizontal PRF to obtain H-PRF gels. Then, a series of studies were carried as following: (a) assessing the weight, solidification and degradation time of H-PRF gels prepared at 45 °C, 60 °C, 75 °C, and 90 °C; (b) investigating the microstructure and rheologic behavior of H-PRF gels prepared at 45 °C, 60 °C, 75 °C, and 90 °C; and (c) evaluating the percentage of live cells in H-PRF gels prepared at 45 °C, 60 °C, 75 °C, and 90 °C using live/dead staining.

## Methods

### Preparation of H-PRF gels (Fig. 1)

**Fig. 1:**
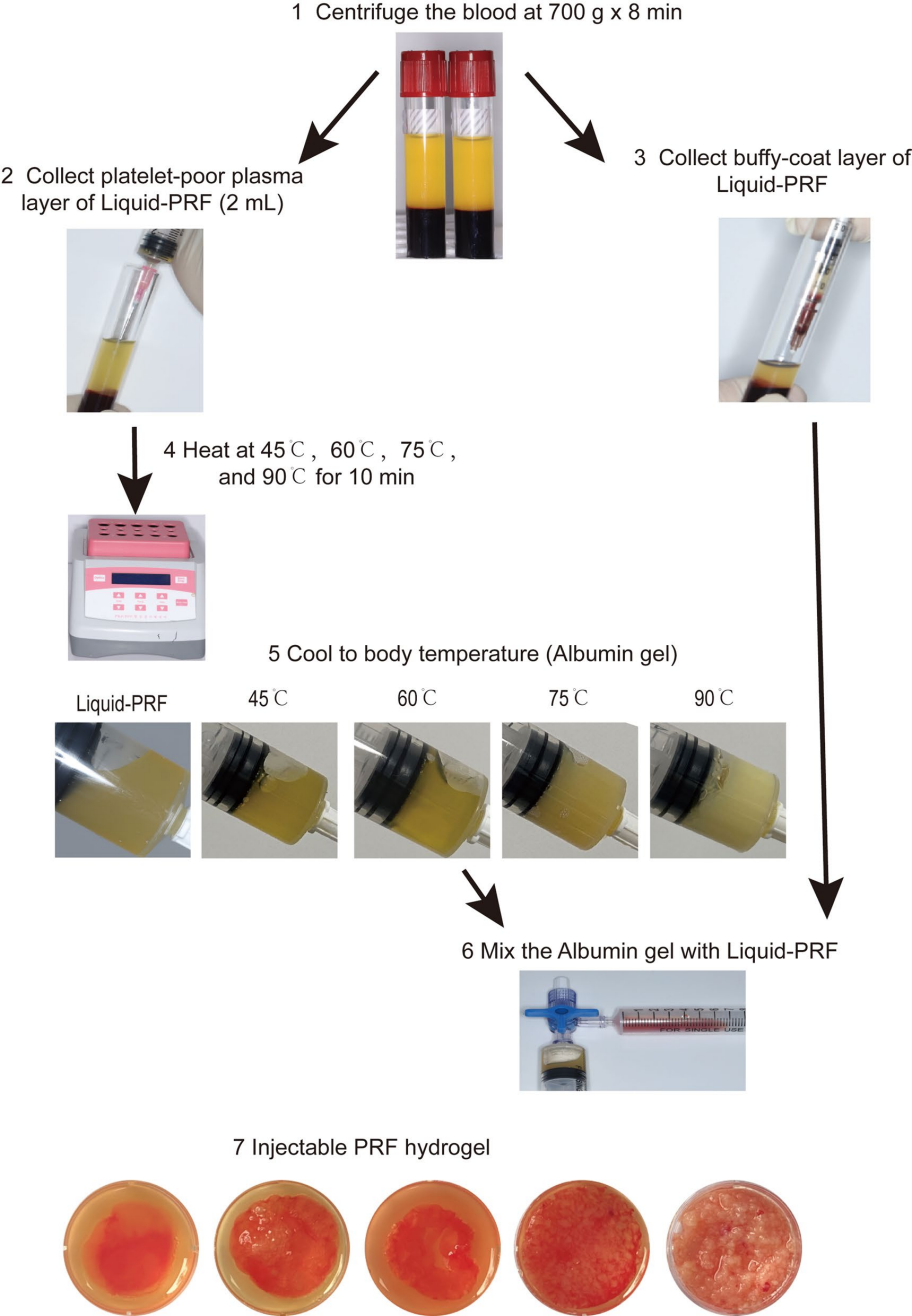
Preparation process of H-PRF gels

All protocols in this study were performed in accordance with the Declaration of Helsinki and approved by the Ethics Committee of the School and Hospital of Stomatology, Wuhan University (B52/2020). Informed consents were obtained from 8 volunteers (average age 26 years). All participants were in good general health as previously described [13]. The inclusion criteria of volunteers are as following: good health, no diabetes, hypertension, heart disease and other systemic diseases; The blood routine indexes were normal and no drugs affecting white blood cells and platelets were taken within 3 months; There is no history of smoking.

In this study, venous blood was collected using plastic PET tubes (Plasmatrident, Weiyin Technology Co., Ltd., Wuhan, China) to prepare liquid H-PRF, the tubes were centrifuged at 700 RCF (*g*) for 8 min at room temperature utilizing a horizontal centrifuge (Bio-PRF, Venice, Florida). After centrifugation, 2-mL upper layer was collected in syringes and heated at 45 °C, 60 °C, 75 °C, and 90 °C for 10 min to create denatured albumin. 4-mL Liquid PRF containing both the upper layer and the buffy coat layer without additional treatment were used as control group. Following heating treatment, the albumin gel was allowed to cool to room temperature for 10 min. Then, the remaining 2-mL liquid H-PRF including remaining cells and growth factor found within the buffy coat layer was mixed with the cooled albumin gel to form H-PRF gel using a female–female luer lock connector. This combination allowed both the lower resorption properties of the albumin gel along with the higher cell content and growth factor content of the liquid H-PRF layer to be thoroughly mixed to form injectable H-PRF gels, the H-PRF gels were then transferred into six-well cell culture plates for future tests [9, 11]. Then, each H-PRF gel was weighed.

### Solidification time

H-PRF gels from each temperature group were also put in six-well cell culture plates immediately after preparation, then the six-well plates were tilted at 45 degrees every 15 s to observe whether the H-PRF gel would dissolve/flow. If the H-PRF gel wouldn’t dissolve, the mixture was deemed to be completely solidified and a corresponding solidification time was recorded as previously described [14].

### Degradation property of H-PRF gel

The H-PRF gel from each group was placed in separate wells of six-well cell culture plates, covered in DMEM and incubated at 37 °C in a humidified atmosphere with 5% CO2 for 23 days. Photos and weighs of the H-PRF gel were recorded every day. The photos revealed the ability for the H-PRF gel to either stay intact or dissolve over time [14, 15].

### Scanning electron microscopy (SEM)

H-PRF gel from each group were fixed with 2.5% glutaraldehyde in 0.1 M sodium cacodylate buffer to fix the H-PRF gel from each temperature for 24 h, and then dehydrated in ascending dilutions of ethanol (50%, 70%, 80%, 95%, and 100%) to dehydrate them. And then, H-PRF gels were critical-point-dried. Finally, the samples were coated with gold automatically. The surface of each group was then captured by SEM (VEGA 3 LMU, TESCAN, Brno, Czech Republic) and investigated for morphological differences [11, 14].

### Rheologic measurements

Rheological measurements of H-PRF gels were carried out to evaluate the mechanical behavior of them. The rheological properties of the gels were measured using a stress-controlled rheometer (Kinexus ultra +, Malvern Instrument Ltd.UK) with a parallel plate (PP50, Ф = 50 mm; the gap was set to 1 mm). The viscosity of gels was determined through the single shear rate test at the shear rate of 0.1 s. To determine the linear viscoelastic region of the gel, an oscillating strain was scanned for the strain of 0.1–10%. To determine the gel failure point of the gel, the amplitude sweep strain (frequency = 1 Hz) was scanned, and the strain scanning range was 0.1–1000%. The strain value of the gel linear viscoelastic zone (strain = 1%) was selected for dynamic frequency scanning, the frequency scanning range was 0.1–10 Hz, and test temperature was 25 °C.

### Live/dead staining

Live/Dead staining was utilized as a tool to evaluate the presence of viable cells within the H-PRF gels. H-PRF gel from each group was put in six-well plates immediately after preparation for evaluating the cell viability using a live-dead staining assay. Live cells were stained with 2 µmol/L Calcein–AM (40747ES80; Yeasen) and dead cells were stained with 4 µmol/L propidium lodide (40747ES80; Yeasen). The cells were washed with PBS and Live/Dead reagents were added and incubated for 1 h at 37 °C. Fluorescent images were captured with a Confocal microscope (Olympus Co., Tokyo, Japan). Thereafter, cell viability was calculated as percentages of live versus total cells.

### Statistical analysis

GraphPad Prism software8.0 (La Jolla, CA) was utilized to analyze the data by analysis of variance (ANOVA). Data are graphed as mean ± SD. *P < 0.05, **P < 0.01, and ***P < 0.001 are considered statistically significant.

## Results

### H-PRF gels weight and solidification time

**Fig. 2:**
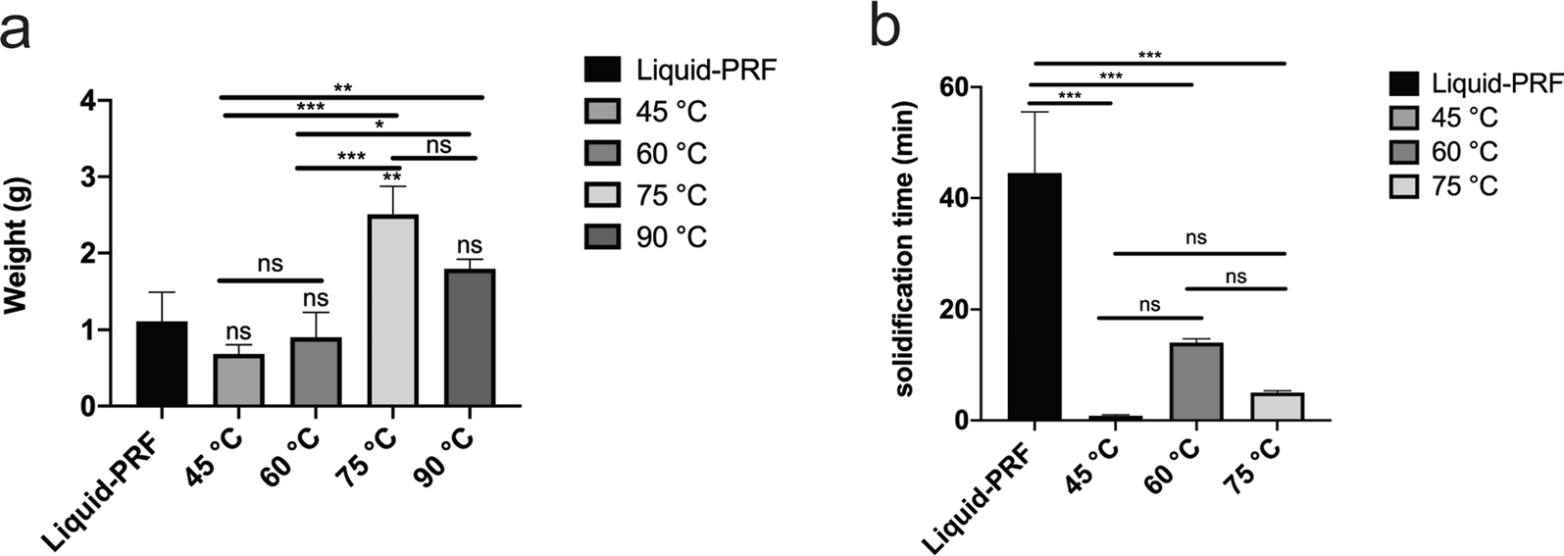
Weight and solidification time of H-PRF gels

As shown in Fig. 2, statistically significant and substantial differences were observed in the weight and solidification time among the 5 different H-PRF gel groups. The H-PRF gel prepared at 75 °C treatment demonstrated the highest weight among all groups, while H-PRF gel prepared at 45 °C treatment demonstrated by far the fastest solidification time into gelated gel when compared to the other groups. The mean solidification time of the normal Liquid-PRF was 44.5 min, whereas the mean solidification time of H-PRF gel prepared at 45 °C, 60 °C, and 75 °C were 0.83 min, 14 min and 5.03 min, respectively. H-PRF gel prepared at 90 °C treatment was unable to form a uniform gel, and the heated part breaks into pieces after the mixing process.

### H-PRF gels degradation property

**Fig. 3:**
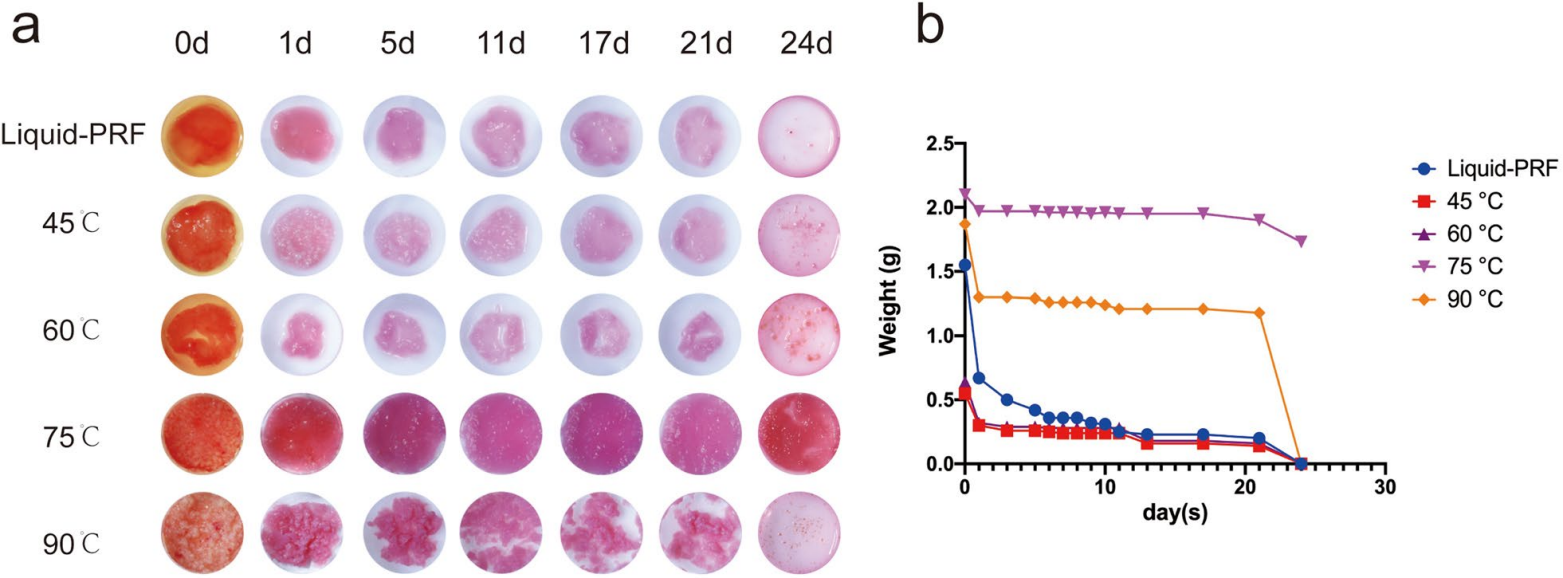
Degradation of H-PRF gels

We evaluated the potential of the H-PRF gel to maintain integrity when immersed in DMEM at 37 °C. The weight of H-PRF gel in each group obtained by different temperatures was reduced along the study period until they were completely degraded after 23 days. After 23 days, control group and the PRF gels group formed under the and prepared at 45 °C and 60 °C was completely degraded, while the PRF gel formed at 75 °C maintained the best integrity, with weight of 1.75 g on the 23rd day (Fig. 3).

### H-PRF gels SEM analysis

**Fig. 4:**
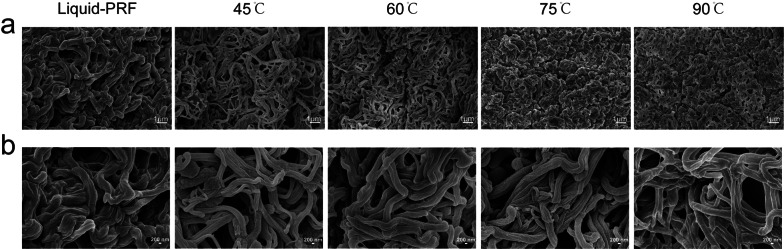
SEM images of H-PRF gels

Ultrastructural evaluation of the H-PRF gels was performed through SEM as shown in (Fig. 4). Interestingly, SEM images revealed that as the temperature increased, while the fibrin bundles seemed thinner, the fibrin network became denser. From the rheologic measurements analysis, we could also find that as the temperature increased, while the fibrin bundles seemed thinner, the mechanical properties of PRF gels became stronger.

### H-PRF gels rheologic measurements analysis

**Fig. 5:**
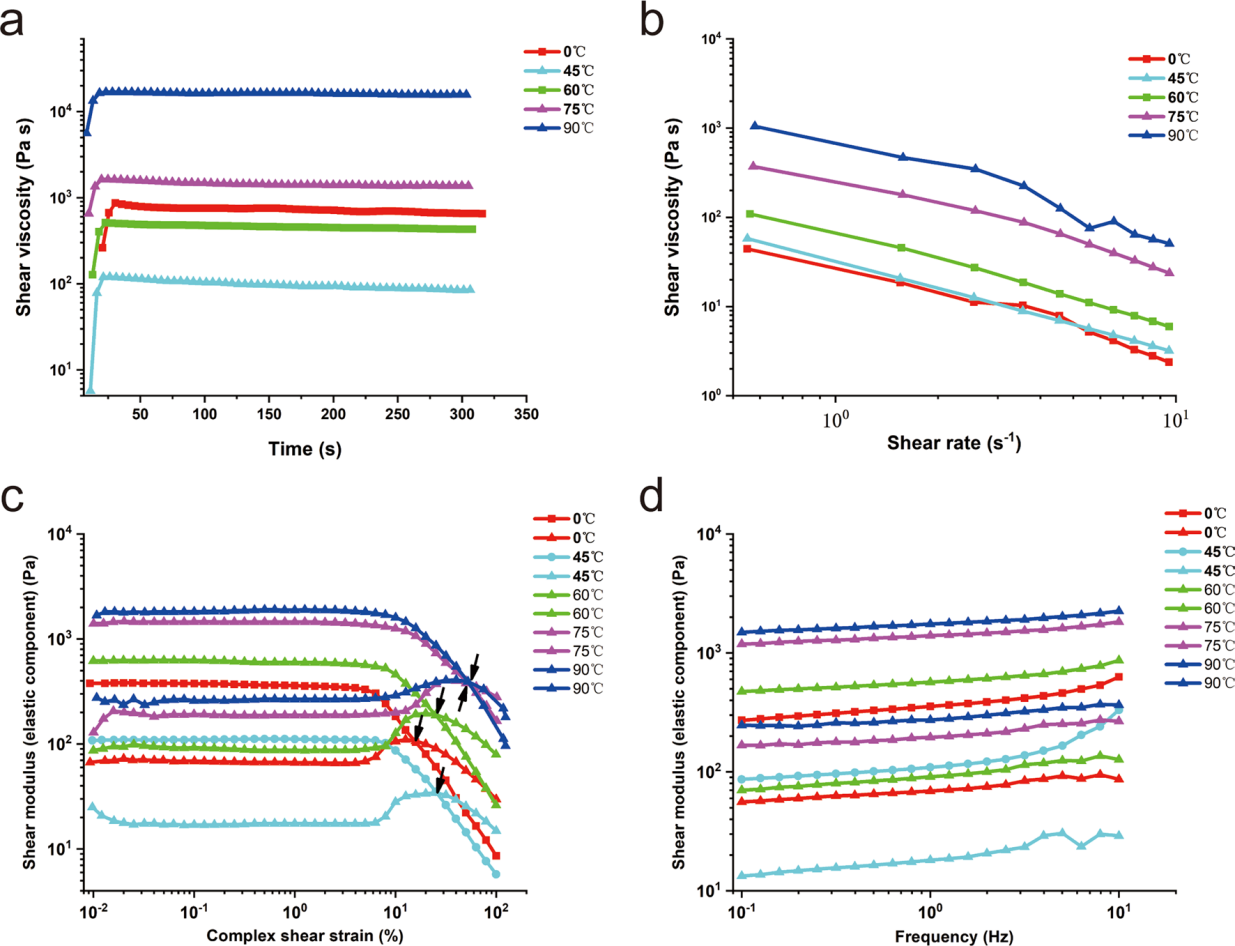
Rheologic behaviors of H-PRF gels

From Fig. 5a, we can found that when the time reached at 200 s, the shear viscosity of the control group is 712 Pa.s, the shear viscosity of the PRF gels heated at 45 °C, 60 °C, 75 °C and 90 °C is 94 Pa.s, 448 Pa.s, 1400 Pa.s and 16,200 Pa.s, respectively. From the results, it can be concluded that the internal three-dimensional network crosslinking of the PRF gels obtained at 45 °C and 60 °C is relatively loose, the internal three-dimensional network of the glue is more and more tightly cross-linked at 75 °C and 90 °C. Figure 5b shows about the shear-thinning rate of PRF gels. The shear viscosity of the control group is 4.65, the shear viscosity of the PRF gels heated at 45 °C, 60 °C, 75 °C and 90 °C is 6.06, 11.47, 38.69 and 111.01, respectively. With the increase of temperature, the cross-linking of PRF became more closely. Figure 5c, d shows about elastic modulus (G′) and viscous modulus (G″) of PRF gels. The results indicated that elastic modulus G′ of PRF gels at different temperatures and in the control group was much larger than the corresponding viscous modulus G″, and G′ and G″ basically changed linearly within 0.1–10 Hz, indicating that the internal cross-linking of PRF formed a stable three-dimensional network structure. In addition, with the increase of temperature, the cross-linking of PRF became more closely. Figure 5 shows about strain of PRF gels. When the strain reaches a certain value, the elastic modulus G′ and the viscous modulus G″ intersect and transform, which indicates that the internal structure of the PRF has been destroyed. When the strain reaches at 15.9%, 25.1%, 25.2%, 50.3%, and 52.4%, the control group, and the PRF gels heated at 45 °C, 60 °C, 75 °C, and 90 °C was damaged, respectively. Compared with other temperatures, the PRF gels prepared at 75 °C and 90 °C requires greater external force to be destroyed, which suggested that with the increase of temperature, the cross-linking of PRF became more closely.

### H-PRF gels cell viability

**Fig. 6:**
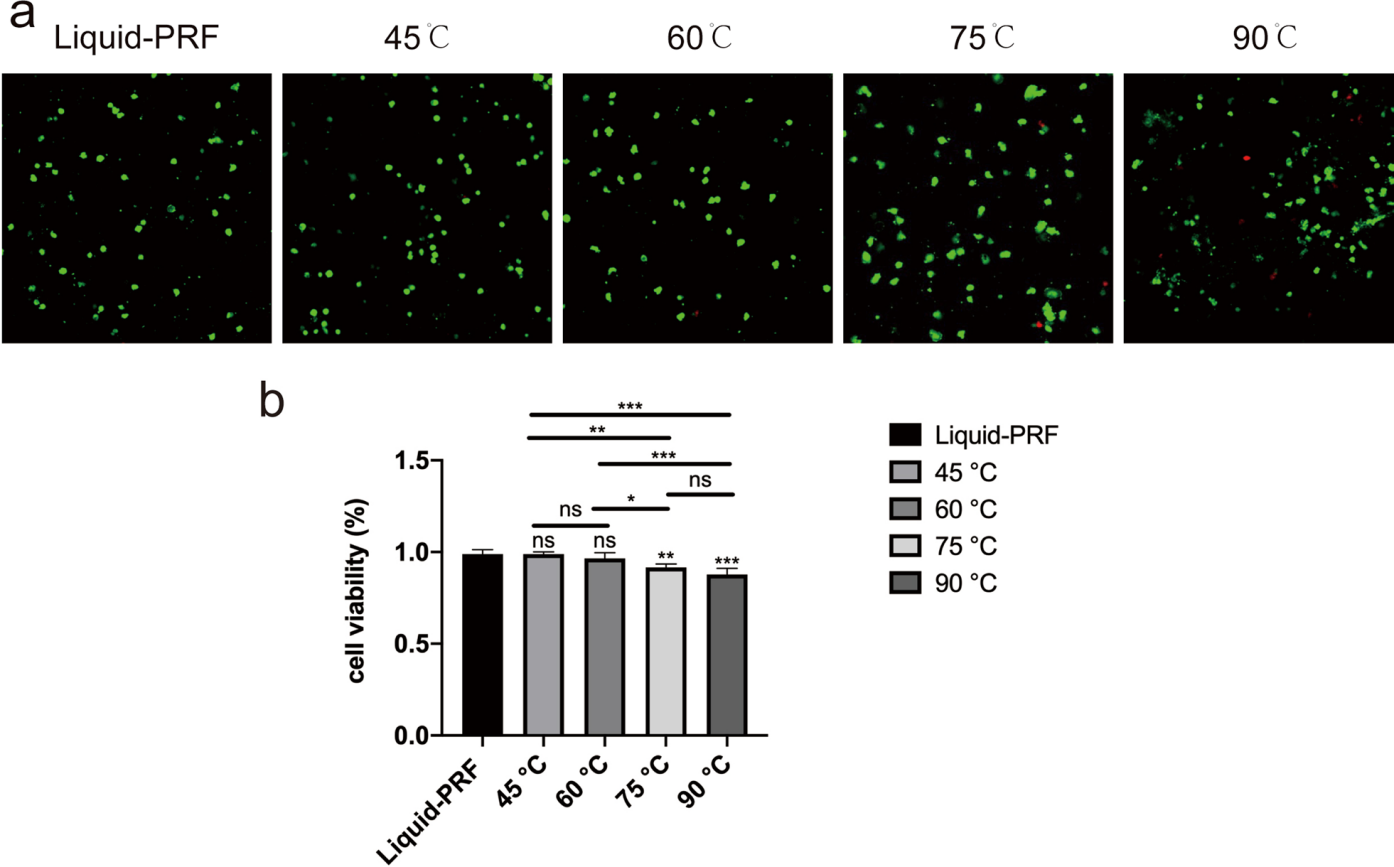
Cell viability within H-PRF gels

The effects of heat treatment on cell viability within the H-PRF gels were studied. As the temperature increased, the number of dead cells increased accordingly. However, regardless of the heating temperature, the number of living cells in H-PRF gels in all groups is greater than 90% (Fig. 6).

## Discussion

In the past 10 years, the use of autologous blood concentrates, such as platelet-rich plasma (PRP) and platelet-rich fibrin (PRF), has become a cosmetic material for skin irritation, enhancement, and rejuvenation [16–19]. A large number of studies (observations, in vitro, animal models and clinical trials) have shown that the local and injection application of platelet concentrate has a significant effect on cell changes and facial regeneration [20–26]. This is mainly because compared with exogenous growth factors and biodegradable material, using autologous platelet growth factors in facial rejuvenation is considered a natural way of regeneration to restore skin degradation. In addition, platelet preparations, besides as the expansion effect of other artificial fillers, will release a lot of growth factor after activation cytokines and extracellular matrix proteins, such as fibrin, fibronectin and polypenins, proteins that bind to specific cell receptors and enhance or modify various intracellular processes involved in cell proliferation and the production of additional extracellular matrix proteins [22, 27, 28].

An ideal esthetic filler should be easy-to-use, long-lasting, biocompatible, non-allergenic, non-carcinogenic, non-migrating, and inexpensive [29]. While blood concentrates does offer greater regenerative potential, it degrades quite rapidly in vivo, within the first 2–3 week post-implantation [6]. For these reasons, many authors have recommended to heat the liquid-PRF to obtain the PRF gel to prolong the degradation time [9, 11]. At the meantime, an appropriate injectable filler should have proper solidification time for handle, and the rheologic properties should be suitable for injection and sustain for volume augmentation.

Gheno et al. proposed to heat PPP layer to extend the degradation time and improve stability of PRF [11]. Nonetheless, it was suggested to verify the cell viability and growth factor activity in PRF after heat treatment. It was reported that heated PRF membranes maintained the growth factor release and favored fibroblast migration, proliferation and collagen deposition. In 2020, Fujioka-Kobayashi et al. reported that PRF retained slow and gradual release of growth factors after heat treatment [9], while Gheno et al. proposed that heated PRF remained volume-stable over 21 days in an animal study [11]. Although the heat treatment protocol was 75 °C for 10 min in current references, the reason for choosing this temperature remained unexplored. Meanwhile, other properties including solidification time, rheologic measurements and cell viability of heated PRF which are essential for injection application have not been reported in previous studies. The aim of the present study was to discover and confirm the optimum temperature for obtaining PRF gel, as well as to evaluate their rheological properties.

This study demonstrated that H-PRF gel obtained at 75 °C for 10 min could form an ideal gelated clot. The weight of H-PRF gel in 75 °C group is the heaviest compared with other temperatures. Instead, it was found that the H-PRF gel obtained at 45 °C for 10 min formed by far the fastest solidification (about 0.8 min). While the H-PRF gel obtained at 75 °C for 10 min was the second fastest solidification (about 5 min), which is suitable for clinical injection operation. The PRF gel obtained by 75 °C for 10 min further demonstrated an ability to resist degradation by showing a much longer degradation time when compared to the other groups. Furthermore, the longer degradation time, denser microstructure and better rheologic properties of the PRF gel prepared via the 75 °C for 10 min approach appears to indicate a greater ability in maintaining the defect volume and stability of graft during facial volume augmentation to ensure satisfactory clinical outcomes. Li et al. found that fibrin fiber stiffness is strongly affected by fiber diameter. In addition, they discovered that fiber modulus, strongly decreased with increasing fiber diameter. In the thin fibers can be 100 times stiffer than thick fibers [30]. From the rheologic measurements analysis, we could also find that as the temperature increased, while the fibrin bundles seemed thinner, the mechanical properties of PRF gels became stronger.

In this study, the percentage of live cells of each group of H-PRF gel in each group was compared in vitro. Through live/dead staining results, we could find that as the temperature increases, the number of dead cells increases. However, regardless of the heating temperature, the number of living cells in PRF gels were greater than 90%. This needed to be done while ensuring the retention of cells from the buffy coat layer and using growth factors to maintain the regenerative properties of PRF within the heated PPP layer.

There are much reports about the application of albumin in tissue engineering [31], as albumin is abundant in serum and easy to obtain. Albumin addition in biomaterials slowed the degradation in vitro [31]. What’s more, it was reported that the combination of albumin and fibrin can help modulating the biomaterial’s ultrastructure and fiber thickness [32]. Heat treatment can modify the secondary structure of albumin, favoring an improvement of its resorption properties and stability [33]. Heating treatment can not only coagulates albumin, but also influence the growth factors in the PRF [34]. PRF is rich in TGF-β, a growth factor important for activation of fibroblasts [35]. Heating treatment of PRF might be two-sided, heating can improve the activity of TGF-β by affecting binding to the latent TGF-β binding protein [36], while temperature over 90 °C would cause TGF-β denaturation [37]. In 2020, Kargarpour et al. found that PPP comprise a TGF-β activity, but the activity is affected by temperature. Thus, the heated PPP needs to be mixed with liquid-PRF to reconstruct TGF-β activity in PRF gel [38].

In view of these data, the association with denatured serum albumin could represent a possible improvement in the PRF-based framework, which is a totally autologous, biocompatible and possibly more durable material with a longer duration of action. However, the growth factor release was not evaluated in this study, and the in vivo degradation of H-PRF gels should also be verified in future study.

## Conclusions

Our results demonstrated that the H-PRF gel obtained by heating upper layer at 75 °C for 10 min could produce a uniform, moldable clot with a longer degradation time and better rheologic behavior with high percent of living cells in H-PRF gel, which is in accordance with previous reports about CGF and fixed angle PRF [9, 11, 39, 40]. It showed promise in facilitating tissue regeneration for facial or dental application. However, future studies including growth factor release and in vivo experiments are needed to further support the clinical application of this heated H-PRF gel in tissue regeneration.

## Data Availability

The data sets used and/or analyzed during the current study available from the corresponding author on reasonable request.
